# PhyloPat: phylogenetic pattern analysis of eukaryotic genes

**DOI:** 10.1186/1471-2105-7-398

**Published:** 2006-09-01

**Authors:** Tim Hulsen, Jacob de Vlieg, Peter MA Groenen

**Affiliations:** 1 Centre for Molecular and Biomolecular Informatics (CMBI), Nijmegen Centre for Molecular Life Sciences (NCMLS), Radboud University Nijmegen, Nijmegen, The Netherlands; 2 Molecular Design and Informatics, NV Organon, Oss, The Netherlands

## Abstract

**Background:**

Phylogenetic patterns show the presence or absence of certain genes or proteins in a set of species. They can also be used to determine sets of genes or proteins that occur only in certain evolutionary branches. Phylogenetic patterns analysis has routinely been applied to protein databases such as COG and OrthoMCL, but not upon gene databases. Here we present a tool named PhyloPat which allows the complete Ensembl gene database to be queried using phylogenetic patterns.

**Description:**

PhyloPat is an easy-to-use webserver, which can be used to query the orthologies of all complete genomes within the EnsMart database using phylogenetic patterns. This enables the determination of sets of genes that occur only in certain evolutionary branches or even single species. We found in total 446,825 genes and 3,164,088 orthologous relationships within the EnsMart v40 database. We used a single linkage clustering algorithm to create 147,922 phylogenetic lineages, using every one of the orthologies provided by Ensembl. PhyloPat provides the possibility of querying with either binary phylogenetic patterns (created by checkboxes) or regular expressions. Specific branches of a phylogenetic tree of the 21 included species can be selected to create a branch-specific phylogenetic pattern. Users can also input a list of Ensembl or EMBL IDs to check which phylogenetic lineage any gene belongs to. The output can be saved in HTML, Excel or plain text format for further analysis. A link to the FatiGO web interface has been incorporated in the HTML output, creating easy access to functional information. Finally, lists of omnipresent, polypresent and oligopresent genes have been included.

**Conclusion:**

PhyloPat is the first tool to combine complete genome information with phylogenetic pattern querying. Since we used the orthologies generated by the accurate pipeline of Ensembl, the obtained phylogenetic lineages are reliable. The completeness and reliability of these phylogenetic lineages will further increase with the addition of newly found orthologous relationships within each new Ensembl release.

## Background

Phylogenetic patterns show the presence or absence of certain genes or proteins in a set of species. These patterns can be used to determine sets of genes or proteins that occur only in certain evolutionary branches. The use of phylogenetic patterns has been common practice as increasing amounts of orthology data have become available. One example is Clusters of Orthologous Groups (COG) [1] which included a Phylogenetic Patterns Search (PPS) on its web interface. This phylogenetic pattern tool was further enhanced with the Extended Phylogenetic Patterns Search (EPPS) [2] tool, providing the possibility of querying the phylogenetic patterns of the COG protein database using regular expressions. The newest release of the OrthoMCL database, OrthoMCL-DB [3], also offers this possibility. However, suchs tool have only been available for querying proteins, and not for genes. The advantage of looking at gene families instead of protein families, is that the view on expansions and deletions is not distorted by any alternative transcripts and splice forms. The PhIGs [4], Hogenom [5] and TreeFam [6] databases all offer phylogenetic clustering of genes, but do not have the functionality of phylogenetic patterns. Here we introduce a web tool named PhyloPat that creates the possibility of querying all complete genomes of the highly reliable Ensembl [7] database using any phylogenetic pattern.

## Construction & content

**Figure 1:**
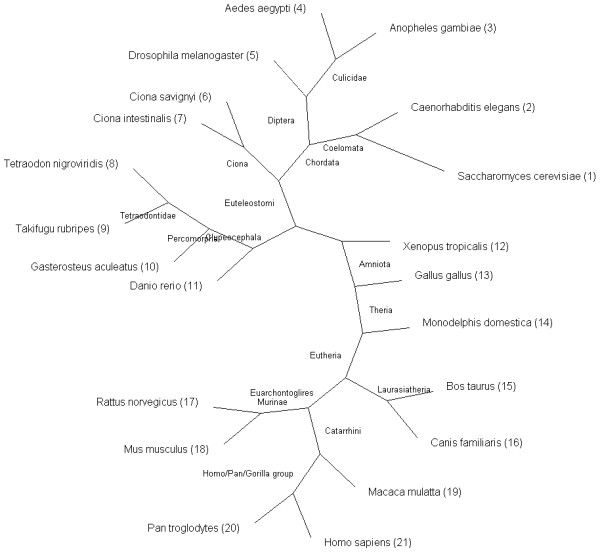
**Phylogenetic tree of all species present in PhyloPat**. This is the unrooted NCBI Taxonomy tree of all species available in Ensembl and PhyloPat. The numbers are the order in which the species are shown on the PhyloPat results pages. A phylogram version of this tree is available through the website.

**Figure 2:**
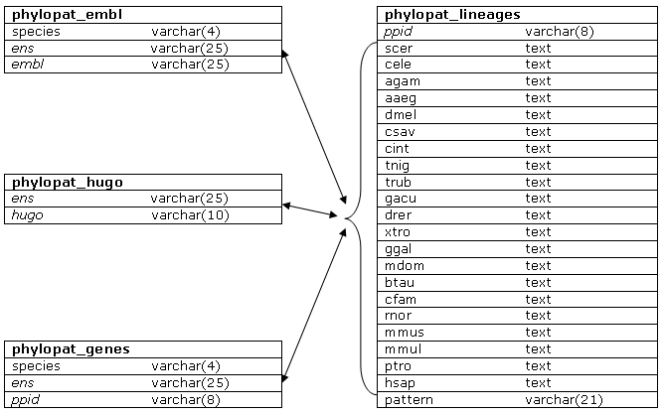
**The PhyloPat database scheme**. The database scheme shows all four tables used in the application. Table names are in bold, primary keys are in italic. Links between fields are shown with arrows. The left side of each column shows the field names, the right side shows the field types.

We generated a set of phylogenetic lineages containing all of the genes in Ensembl [7] that have orthologs in other species according to the EnsMart [8] database. This set covers all of the 21 (eukaryotic) species available in EnsMart version 40 (pre-versions and low coverage genomes not taken into account). We collected the complete set of orthologies between these species: 420 species pairs, 446,825 genes and 3,164,088 orthologous relationships. These orthologies consist of 2,000,706 one-to-one, 795,723 one-to-many and 367,659 many-to-many relationships, created by the very extensive orthology prediction pipeline [9] from Ensembl. This pipeline starts with the collection of a number of Best Reciprocal Hits (BRH, proven to be accurate [10]) and Best Score Ratio (BSR) values from a WUBlastp/Smith-Waterman whole-genome comparison. These are used to create a graph of gene relations, followed by a clustering step. These clusters are then applied to build a multiple alignment using MUSCLE [11] and a phylogenetic tree using PHYML [12]. Finally, the gene tree is reconciled with the species tree using RAP [5]. From each reconciled gene tree, the above mentioned orthologous relationships are inferred. After the collection of all orthologous pairs, we generated phylogenetic lineages using a single linkage algorithm. First, we determined the evolutionary order of the studied species using the NCBI Taxonomy [13] database. The phylogenetic tree of these species, together with some phylogenetic branch names, can be seen in Figure 1. Second, we used this phylogenetic tree as a starting point for building our phylogenetic lineages. For each gene in the first species (*S. cerevisiae*), we looked for orthologs in the other 20 species. All orthologs were added to the phylogenetic lineage, and in the next round were checked for orthologs themselves, until no more orthologies were found for any of the genes. This process was repeated for all genes in all 21 species that were not yet connected to any phylogenetic lineage yet. The complete phylogenetic lineage determination generated 147,922 lineages. Please note that the phylogenetic order that we have determined here does not affect the construction of the phylogenetic lineages in any way: changing the order only influences the numbering of the phylogenetic lineages but not the contents of the lineages. This is due to our clustering method, in which each orthologous relationship is treated symmetrically. Figure 2 shows the database scheme: the phylogenetic lineages and some extra information have been stored in four tables, optimized for fast querying.

## Utility & discussion

### Utility

**Figure 3:**
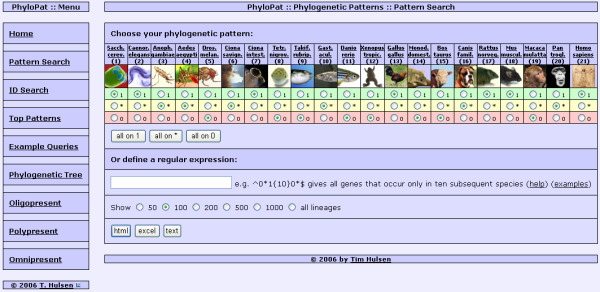
**The PhyloPat web interface (Pattern Search tab)**. The web interface has the menu on the left and the input/results page on the right. On the pattern search page, the user can generate a phylogenetic pattern by clicking a radio button for each species. 1 = present, * = present/absent, 0 = absent. The buttons directly below put all 21 species on the corresponding mode. MySQL regular expressions offer the possibility of advanced querying. The user can choose to show any number of lineages and choose the output format: HTML, Excel or plain text.

We developed an intuitive web interface (Figure 3) named PhyloPat to query a MySQL database containing these phylogenetic lineages and derived phylogenetic patterns. As input a phylogenetic pattern is used, generated by clicking a set of radio buttons or by typing a regular expression, or a list of Ensembl or EMBL identifiers. The application of MySQL regular expressions provides enhanced querying. The output can be given in HTML, Excel or plain text format. A link to the FatiGO web interface has been incorporated in the HTML output, creating easy access to functional information. Each phylogenetic lineage can be viewed separately by clicking the PhyloPat ID (PPID). This view gives all Ensembl IDs within the phylogenetic lineage plus the HUGO [14] gene names. The web interface also provides some example queries, the 100 most occurring patterns, and numerical overviews of lineages that are present in 1) all species 2) almost all species and 3) only one or two species. Finally, a phylogenetic tree of all included species is provided, through which each branch can be selected to view a list of branch-specific genes. This tree can be downloaded in PHYLIP [15] format.

### Omnipresent genes

**Figure 4:**
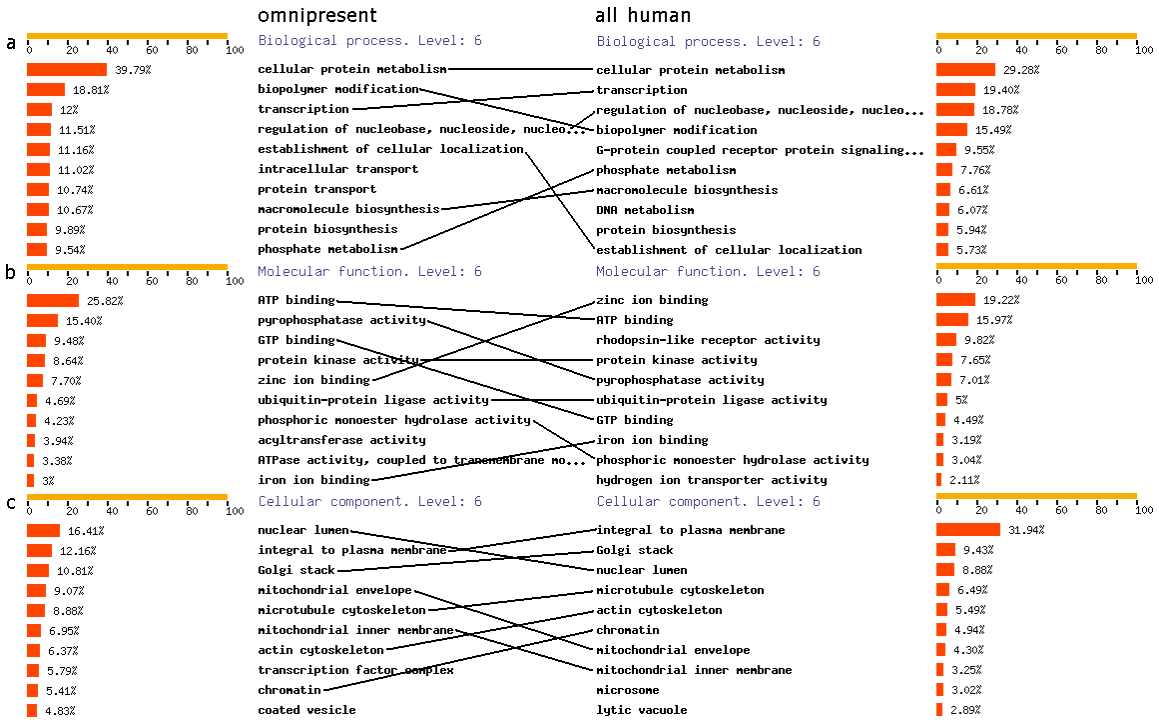
**Gene Ontology annotations of 1) omnipresent and 2) all human genes**. The left side shows the Gene Ontology annotations for all 2,185 human genes in omnipresent lineages. The right side shows the Gene Ontology annotations for all 31,718 human genes, used as a reference set. Lines are placed between equal annotations for easy comparisons between the left and the right side. **a.) **6^th ^level GO Biological Processes. **b.) **6^th ^level GO Molecular Functions. **c.) **6^th ^level GO Cellular Components.

An analysis of all lineages with the phylogenetic pattern '111111111111111111111' (or MySQL regular expression '^1+$') gives a list of 'omnipresent' genes, i.e. present in all 21 species. We found 1001 omnipresent genes, which are most likely involved in important functions, since they are present in all species. Figure 4 shows the GO annotation [16] for all 2185 human genes within these omnipresent phylogenetic lineages, generated by FatiGO [17]. When human genes are present in the output, FatiGO can be queried by clicking a button below the output. To compare the results, we also show the GO annotation for the complete set of human genes (31,718 in Ensembl v40). Lines are drawn between similar GO classifications, to facilitate easy comparison between the omnipresent genes and all human genes. It is clear from the 6^th ^level GO biological process annotation (Figure 4a) that omnipresent genes are less often involved in transcription compared to a human gene chosen at random, but more often in cellular protein metabolism and establishment of cellular localization. We suggest that the process of transcription does not need that many genes in the 'lower' species, but in the 'higher' species, like human, many transcription related gene families have expanded ([18], table 1). Analysis of the 6^th ^level GO molecular functions (Figure 4b) shows that many omnipresent genes have ATP binding or pyrophosphatase activity, while the human gene set consists for almost 10% of genes with rhodopsin-like receptor activity. The latter is due to the fact that the GPCR class A family has expanded greatly in mammals ([19], table 2). Finally, the 6^th ^level GO cellular components (Figure 4c) show that a lesser fraction of the omnipresent genes are integral to the plasma membrane.

**Table 1:** Phylogenetic lineages containing human Hox cluster genes

**ppid**	**sc**	**ce**	**ag**	**aa**	**dm**	**cs**	**ci**	**tn**	**tr**	**ga**	**dr**	**xt**	**gg**	**md**	**bt**	**cf**	**rn**	**mm**	**mm**	**pt**	**hs**	**pattern**	**gene**
PP022041	0	1	1	1	1	1	1	3	6	5	6	2	2	3	3	2	3	3	2	2	2	011111111111111111111	MSX1, 2
PP024984	0	0	1	0	0	0	0	1	1	1	1	1	0	0	1	1	1	1	1	1	1	001000011111001111111	C4
PP027791	0	0	1	1	1	0	0	2	3	3	4	3	2	3	3	3	3	3	3	3	3	001110011111111111111	TLX1, 2, 3
PP049478	0	0	0	0	0	0	2	2	1	1	5	3	1	1	2	3	2	2	2	2	3	000000111111111111111	B8, C8, D8
PP053824	0	0	0	0	0	0	0	1	1	1	2	0	0	1	0	1	0	1	0	1	1	000000011110010101011	D11
PP053827	0	0	0	0	0	0	0	2	2	2	1	1	1	1	1	1	1	1	1	1	1	000000011111111111111	A10
PP053828	0	0	0	0	0	0	0	2	1	1	1	1	2	1	2	1	2	2	2	2	2	000000011111111111111	C13, D13
PP053829	0	0	0	0	0	0	0	6	3	3	4	1	1	2	2	2	2	2	2	2	2	000000011111111111111	A1, B1
PP053830	0	0	0	0	0	0	0	1	1	1	1	0	0	1	0	1	1	1	1	1	1	000000011110010111111	B4
PP053832	0	0	0	0	0	0	0	2	1	1	1	1	0	1	1	1	1	1	1	1	1	000000011111011111111	A5
PP053833	0	0	0	0	0	0	0	2	1	1	1	0	1	1	1	1	1	1	0	1	1	000000011110111111011	B2
PP053834	0	0	0	0	0	0	0	3	1	1	0	1	0	1	1	1	1	1	1	1	1	000000011101011111111	D3
PP053835	0	0	0	0	0	0	0	2	1	1	1	0	1	1	1	1	1	1	1	0	1	000000011110111111101	A9
PP053836	0	0	0	0	0	0	0	2	1	1	1	1	1	1	1	1	1	1	1	1	1	000000011111111111111	A3
PP053838	0	0	0	0	0	0	0	2	1	1	1	0	1	0	1	1	1	1	1	1	1	000000011110101111111	C12
PP053839	0	0	0	0	0	0	0	1	1	1	1	1	1	1	1	1	1	0	1	1	1	000000011111111110111	D4
PP053840	0	0	0	0	0	0	0	2	1	1	1	1	2	0	1	0	1	1	1	0	1	000000011111101011101	C11
PP053842	0	0	0	0	0	0	0	4	3	2	2	1	1	1	1	1	1	1	1	1	1	000000011111111111111	A13
PP053844	0	0	0	0	0	0	0	3	2	2	3	1	0	1	1	1	1	1	1	1	1	000000011111011111111	B5
PP053845	0	0	0	0	0	0	0	2	1	1	1	1	1	1	1	1	1	1	0	1	1	000000011111111111011	B3
PP053846	0	0	0	0	0	0	0	2	1	1	2	1	1	1	1	1	1	1	1	1	1	000000011111111111111	D10
PP053847	0	0	0	0	0	0	0	2	2	2	1	1	1	1	1	1	1	1	1	1	1	000000011111111111111	A2
PP053849	0	0	0	0	0	0	0	3	4	1	5	1	1	3	2	3	3	3	3	2	3	000000011111111111111	A6, B6, C6
PP053853	0	0	0	0	0	0	0	1	1	1	0	1	1	1	1	1	1	1	0	1	1	000000011101111111011	A4
PP053854	0	0	0	0	0	0	0	3	2	2	5	2	2	2	3	1	3	3	2	1	3	000000011111111111111	B9, C9, D9
PP053858	0	0	0	0	0	0	0	1	1	1	2	0	0	1	1	1	1	1	1	1	1	000000011110011111111	A11
PP070659	0	0	0	0	0	0	0	0	0	1	2	1	2	1	2	2	2	2	2	2	2	000000000111111111111	A7, B7
PP075622	0	0	0	0	0	0	0	0	0	0	1	0	0	0	1	1	1	1	1	1	1	000000000010001111111	C5
PP084287	0	0	0	0	0	0	0	0	0	0	0	1	1	0	1	1	1	1	1	1	1	000000000001101111111	C10
PP085049	0	0	0	0	0	0	0	0	0	0	0	1	0	1	1	0	1	1	1	1	1	000000000001011011111	D1
PP087941	0	0	0	0	0	0	0	0	0	0	0	0	1	1	1	0	1	1	1	1	1	000000000000111011111	D12
PP089685	0	0	0	0	0	0	0	0	0	0	0	0	1	1	1	1	1	1	1	1	1	000000000000111111111	B13

**Table 2:** Analysis of phylogenetic lineages containing human Hox cluster genes

**ppid(s)**	**name**	**cluster A**	**cluster B**	**cluster C**	**cluster D**	**first sp**.	**position**
PP053829, PP085049	HOX1	HOXA1	HOXB1		HOXD1	T. nigrov.	anterior
PP053847, PP053833	HOX2	HOXA2	HOXB2			T. nigrov.	anterior
PP053836, PP053845, PP053834	HOX3	HOXA3	HOXB3		HOXD3	T. nigrov.	PG3
PP053853, PP053830, PP024984, PP053839	HOX4	HOXA4	HOXB4	HOXC4	HOXD4	A. gamb.	central
PP053832, PP053844, PP075622	HOX5	HOXA5	HOXB5	HOXC5		T. nigrov.	central
PP053849	HOX6	HOXA6	HOXB6	HOXC6		T. nigrov.	central
PP070659	HOX7	HOXA7	HOXB7			G. acul.	central
PP049478	HOX8		HOXB8	HOXC8	HOXD8	C. intest.	central
PP053835, PP053854	HOX9	HOXA9	HOXB9	HOXC9	HOXD9	T. nigrov.	posterior
PP053827, PP084287, PP053846	HOX10	HOXA10		HOXC10	HOXD10	T. nigrov.	posterior
PP053858, PP053840, PP053824	HOX11	HOXA11		HOXC11	HOXD11	T. nigrov.	posterior
PP053838, PP087941	HOX12			HOXC12	HOXD12	T. nigrov.	posterior
PP053842, PP089685, PP053828	HOX13	HOXA13	HOXB13	HOXC13	HOXD13	T. nigrov.	posterior
PP027791	TLX	TLX1	TLX2	TLX3		A. gamb.	
PP022041	MSX	MSX1	MSX2			C. eleg.	

### Oligopresent genes

The distribution of 'oligopresent' genes (genes that exist in only one/two species) can be used to determine which species are evolutionary most related, as the number of shared genes, that are absent in other species, can be used as a measure for the phylogenetic distance [20]. It is apparent that are the closest relatives are *C. savignyi *and *C. intestinalis *(1737 oligopresent genes), followed by *T. nigroviridis *and *T. rubripes *(1572 oligopresent genes) and *A. gambiae *and *A. Aegypti *(1058 oligopresent genes). These results correspond perfectly with the current opinion on evolutionary relationships. It should also be noted that the number of genes present in only one species is this high because of the incomplete orthology information contained in the EnsMart database. This will improve with each new Ensembl release, as orthology information and functional annotation are expanded in each release.

### Polypresent genes

A second measure for evolutionary relatedness is the distribution of 'polypresent' genes: genes that are missing in only one or two species. *S. cerevisiae *has the highest number of missing polypresent genes: 961 polypresent genes do not occur in *S. cerevisiae *only, and 854 polypresent genes are not present in *S. cerevisiae *and a second species. Other high-scoring pairs include both *Ciona *species (47 absent polypresent genes) and the combination of one of these *Ciona *species with *G. gallus *(16 and 14 absent polypresent genes). The relatively high number for the latter pair is striking, because these species are not closely related. One would suspect such a high number only for two species that are relatively closely related, which is the case for the two *Ciona *species.

### Case study: Hox genes

As a case study we used the highly researched and from an evolutionary point-of-view very interesting Hox genes. First, we searched the Ensembl database for human genes with the term 'hox' in the annotation. We found 44 genes, which were entered into PhyloPat. The output is shown in Table 1. The lists of Ensembl IDs have been replaced by the number of IDs. 32 phylogenetic lineages were found, one of which were already present in *C. elegans*: PP022041. This lineage contains the Msh homeobox-like proteins. PP024984 and PP027791, containing the HOXC4 and TLX lineages, are only found in the Coelomata: *A. gambiae *and further. No less than 22 lineages originated in the early vertebrates, presented by *T. nigroviridis*. HOXD12 and HOXB13 are only present in mammals.

Striking observations can be made with the fish species: all three species have significantly more Hox genes than the mammals. *T. nigroviridis*, for example, has 57 genes in this lineage, while *M. domestica *has only 35. These numbers correspond well with the fact that Teleost fish have at least seven Hox clusters, whereas mammals have only four [21]. Mammals also have less Hox genes per cluster, demonstrating that there has been gene loss within the Hox clusters since the evolution from a vertebrate ancestor to present-day mammals [22]. Table 2 shows the further analysis of the Hox genes using the PhyloPat output. *H. sapiens *misses the genes HOXA8, HOXB10, HOXB11, HOXC1, HOXC2, HOXC3, HOXC7, HOXD2, HOXD5, HOXD6 and HOXD7. The absence of these 11 genes is in agreement with current knowledge of human Hox genes (figure 3A of [22]). Two exceptions exist: HOXC8 instead of HOXC7, and the absence of HOXA12. The HOXA12 gene cannot be found in the other mammals either.

### Functional annotation

PhyloPat can be used for annotation of genes with unknown functions. When a gene with unknown function is clustered in a certain phylogenetic lineage, the function of other genes in that lineage can be assigned to the gene with unknown function. For example, the PP001723 lineage [23] contains a number of genes that have an unknown function, under which the ENSANGG00000008970 gene from *A. gambiae *and the ENSCING00000000880 gene from *C. intestinalis*. By using the orthology information provided by Ensembl and the PhyloPat clustering into one lineage, we can see that all of these genes are connected to the human gene KLHDC4. This function can now be assigned to the genes with unknown function.

### Discussion

The above examples show that PhyloPat is useful in evolutionary studies and gene annotation. It continues on the concept of phylogenetic pattern tools like EPPS [2], and on gene databases like TreeFam [6] and Homogen [5]. The originality of PhyloPat lies in the combination of these two aspects: phylogenetic pattern querying and gene family databases. In PhyloPat it is possible to determine a species set that should be included (1), a species set that should be excluded (0) and a species set which presence is indifferent (*). This, and the use of regular expression queries, enables quite complicate phylogenetic patterns searches and clustering. For example, with PhyloPat it is quite easy to find two sets of genes that have completely anti-correlating patterns (like '001111100011000000000' and '110000011100111111111'). Some of these genes from the different sets might turn out to be analogous, i.e. performing the same function but having different ancestor genes. Such kind of analysis is very hard to do with TreeFam or Hogenom. Furthermore, we aim to provide an easy-to-use web interface in which the Ensembl database can be queried using phylogenetic patterns. In just one second, users can see which gene families are present in a certain species set but missing in another species set. The output of our application can be easily analyzed by the FatiGO tool, like we demonstrated in figure 4. Finally, PhyloPat has the advantage of only relying on the Ensembl database. Treefam and Hogenom use a wide range of gene and protein databases, each with their own standards and methodologies. By using only the Ensembl database (considered by many to be the standard genome database) as input, we create a non-redundant database, through which it is possible to easily study lineage-specific expansions of gene families.

## Conclusion

The analyses of the oligopresent, polypresent and omnipresent genes, as well as the small case study of the Hox genes, are just a few examples of what can be done with phylogenetic patterns in general and PhyloPat in particular. Using this tool, it is easy to find genes that e.g. occur for the first time in vertebrates, occur only in a specific number of species, or are unique for a certain species. It will be of help in the annotation of genes with unknown functions. By comparing the genes in lineages with anti-correlating patterns, it will also help finding analogous genes. PhyloPat will be completely recalculated with each major Ensembl release to ensure up-to-date and reliable phylogenetic lineages.

## Availability & requirements

PhyloPat is freely available at .

## Abbreviations

BRH Best Reciprocal Hit

BSR Best Score Ratio

COG Clusters of Orthologous Groups

EMBL European Molecular Biology Laboratory

EPPS Extended Phylogenetic Patterns Search

GO Gene Ontology

HUGO HUman Genome Organisation

MCL Markov Cluster Algorithm

MUSCLE MUltiple Sequence Comparison by Log-Expectation

MySQL My Structured Query Language

PHYLIP PHYLogeny Inference Package

PHYML PHYlogenetic reconstruction by Maximum Likelihood

PPS Phylogenetic Patterns Search

## Authors' contributions

TH carried out the construction of the phylogenetic lineages, designed the database structure, built the website, and drafted the manuscript

JdV participated in the design of the study

PG participated in the design and coordination of the study and helped to draft the manuscript

All authors read and approved the final manuscript
